# Explaining Lengths and Shapes of Yeast by Scaling Arguments

**DOI:** 10.1371/journal.pone.0006205

**Published:** 2009-07-10

**Authors:** Daniel Riveline

**Affiliations:** 1 Laboratory of Yeast Genetics and Cell Biology, The Rockefeller University, New York, New York, United States of America; 2 Laboratoire de Spectrométrie Physique (CNRS), UMR 5588, Université Joseph Fourier, Saint-Martin d'Hères, France; University of East Piedmont, Italy

## Abstract

Lengths and shapes are approached in different ways in different fields: they serve as a read-out for classifying genes or proteins in cell biology whereas they result from scaling arguments in condensed matter physics. Here, we propose a combined approach with examples illustrated for the fission yeast *Schizosaccharomyces pombe.*

## Introduction

Cells are regulated by highly connected signalling pathways [1]: activation and inhibition cascades are constantly changing the cell responses to its environment and to its own dynamics. In order to isolate independent signalling modules, there is a requirement to identify simple and reliable readouts. Levels of molecular activity such as proteins phosphorylation and dephosphorylation are efficient for this purpose. However microscopic cellular lengths and shapes have also been proven to be powerful readouts for classifying networks in cellular control. For example, genes deletions lead to classes of strains having different lengths [2] and modified shapes [3]. Genes leading to a similar phenotype are then grouped into a functional biological module.

Similar microscopic measurements are usually treated by *scaling* arguments in condensed matter physics. Key parameters of the system are extracted, and lengths or shapes formulae are derived using appropriate combinations of parameters. This approach has proven its efficiency for a variety of systems, ranging from whole organisms [4] to polymer physics [5] and wetting phenomena [6]. Since the selected parameters have to completely capture the matter properties of the system under study, these scaling laws reflect the physical relations bound to the problem. As a result, these laws provide satisfactory physical explanations for the measured lengths and shapes, beyond the fact that the derived formulae are constrained by the dimensional analysis of the parameters units. In addition, these scaling laws allow to predict changes in lengths and shapes caused by the variations of selected - and often unexpected - parameters.

I propose here to couple both genetic and mesoscopic approaches on a unicellular organism, the fission yeast *S. pombe*. The fission yeast cell is a rod of 15 µm length and 4 µm diameter with a rigid wall. Cells grow by elongation from the hemispherical ends and divide by medial fission. Wall tension and pressure difference between the inside and the outside of the cell are the main physical parameters used for explaining phenotypes [7]. The derived read-outs are here curvature at cell ends, cell radius, cytokinetic ring centering, lengths at “NETO”, C shape (ban mutants, see below). The relations are derived, and data illustrating the results are given; in addition, experiments are suggested for probing the laws in future works. The main contribution of this paper is to propose a quantitative framework to understand the microscopic read-outs, while suggesting new approaches for classifying genes.

## Methods

### Two laws for fission yeast shape

According to the Pascal principle, the difference in pressure between the inside and the outside of the cell is constant
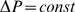
(1)This property imposes a constant *global* pressure around the cell. The force associated with this pressure is perpendicular to the wall.

In contrast, the Young-Laplace equation imposes that *local* surface properties dictate local shapes:
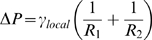
(2)where γ_local_ is the local surface tension, and R_1_ and R_2_ are the principal radii of curvature. This relation states that the pressure force perpendicular to the surface of the cell is balanced by the local elastic properties of the wall. As a result, this equation suggests that the cell shapes are directly set by the global pressure difference and wall local surface tensions.

## Results

### Curvature at the cell ends: a low value for membrane surface tension as the motor for recruiting the growth machinery

**Figure 1:**
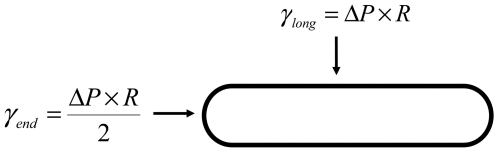
Scaling for lengths during cell extension; the tension γ_local_ is different between the hemispherical ends and the cylindrical longitudinal side (R is the cell radius; ΔP is the pressure difference).

The cell growth machinery assembles at one end of the cell after septation [8]. Key cytoplasmic proteins of this machinery leading to synthesis and local deposition of cell wall material are distributed around the hemispherical end (see for example [9]–[11]). This spatial organisation and the exclusion from the side of the cell long axis are surprising. It is not due to the microtubule cytoskeleton, since the same machinery operates in the absence of microtubules [12]. We propose that surface tension at *the membrane* may explain this preferred location for assembly: following Young-Laplace equation, the tension around the cap is twice lower than the tension along the side of the cell (see Figure 1); the growth machinery is thus preferentially inserted around this hemispherical cap.

We can give an estimate for the membrane tensions. Assuming that yeast membrane lipid composition is similar to mammalian cell membrane, we can use the 10^−4^ N/m tension value measured for fibroblasts (see [13], [14]). We apply this value to the tension at the hemispherical end of the yeast cell. Following our argument, the longitudinal tension is about 2.10^−4^ N/m. Note that we present this estimate as a reasonable order of magnitude. Membrane tension measurements on fission yeast cells without a wall (*cytoplasts* [15]) will be required for confirming this value.

The following shapes mutants are consistent with this surface tension argument. Strains with T shapes have been documented in various conditions: they are obtained either by genetic modifications [12] or by removal of microtubules [16]. These strains exhibit a new growth zone in the side of the cell, with the same radius as regular growing ends: a hemispherical deformation appears which leads to further recruitment of the cell wall machinery; this step is followed by further growth. Additional growth zones appear along the sides of the cell with the same mechanism [16], i.e. local deformation of the cells, followed by elongation. We propose that the local reduced tension promotes the local recruitment of the machinery. Microtubules in wild type cells would restrict the remodelling of the wall exclusively at the ends of the cell; in these T-shaped cells, however, local wall remodelling on the side would trigger the local deformation due to the pushing force of the pressure.

In order to test this result, the following experiments could be performed: (i) decreasing the wall thickness locally by spraying a wall digesting enzyme (see [15]) close to the cell should promote a new local growing end (for the method of local spray, see for example [17]); the pressure will have promoted the local deformation of the cell, followed by the recruitment of the growth machinery; and (ii) forcing the cells into closed microfabricated patterns like in [18] with designed hemispherical ends should alter growth in both ways: a cell end with an imposed curvature smaller than wild type ends should promote growth, whereas an end with a larger curvature should block further cell elongation.

### Estimate of the pressure difference with the use of cell wall tension

We now consider the outer layer of yeast, the cell *wall* and its associated surface tension. Note that this layer is close but distinct from the cell *membrane* mentioned in the previous paragraph. Taking the expressions of the radius on the long axis
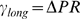
(3)(see Figure 1), we can derive two key features for fission yeast: (i) since the cell diameter is constant during cell growth, pressure difference remains *constant* during cell growth; (ii) we can estimate this pressure difference; surface tension is the product of the wall Young modulus E by the wall thickness w, so
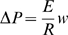
(4)Based on whole cell measurements for E of 100 MPa [19], [20], and taking a wall thickness w of 200 nm [21], we obtain a pressure difference of about 10 MPa. Direct measurements similar to experiments on molds by Money et al [22] should allow to probe this estimate for fission yeast.

### Length at mitosis: the septum location

**Figure 2:**
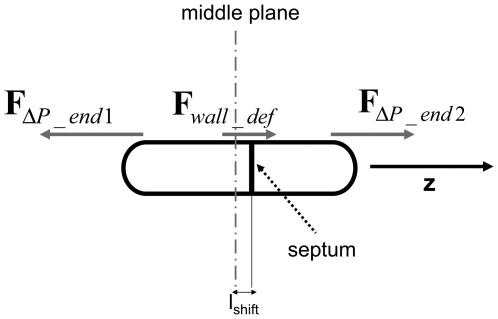
Scaling for the septum location at mitosis; the cytokinetic ring contraction leads to the septum formation; its location is shifted from the cell central plane by a distance lshift. Forces at the cell wall along the z-axis are represented.

When cells reach mitosis, an acto-myosin ring is assembled around the central part of the cell [23]. The contraction of this ring associated with the local addition of cell wall leads to the formation of a septum and to the subsequent separation of sister cells. Strikingly this septum is located in the vicinity of the middle of the cell (see Figure 2). We show here that simple arguments can determine its location.

I propose that the cell is under pressure while no wall is added at this stage of the cycle. The wall is then undergoing a longitudinal deformation: the pressure imposes traction forces at both ends; the wall is deformed along a distance l_ext_ (like a spring being pulled at both ends). We call l_shift_ the distance between the middle of the cell and the location where forces are balanced.

We then should balance forces using the Young-Laplace equation along the z axis (see Figure 2); we have at both ends:
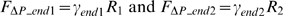
(5)


We assume that the wall elasticity is isotropic. The force associated with the deformation of the wall is given by:
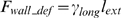
(6)The z-location where forces are balanced is given by:
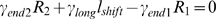
(7)So
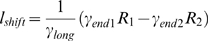
(8)Since
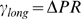
(9)(see Eq. 3), we obtain
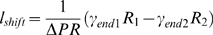
(10)We have
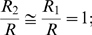
we conclude that the septum location is shifted from the center by the distance l_shift_ given by:
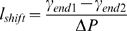
(11)


Qualitatively, it suggests that septa are closer to ends with a larger radius, which is what is experimentally observed in cells with ends of different radii (see for example in [24]).

### New-end take off (NETO) Length

**Figure 3:**
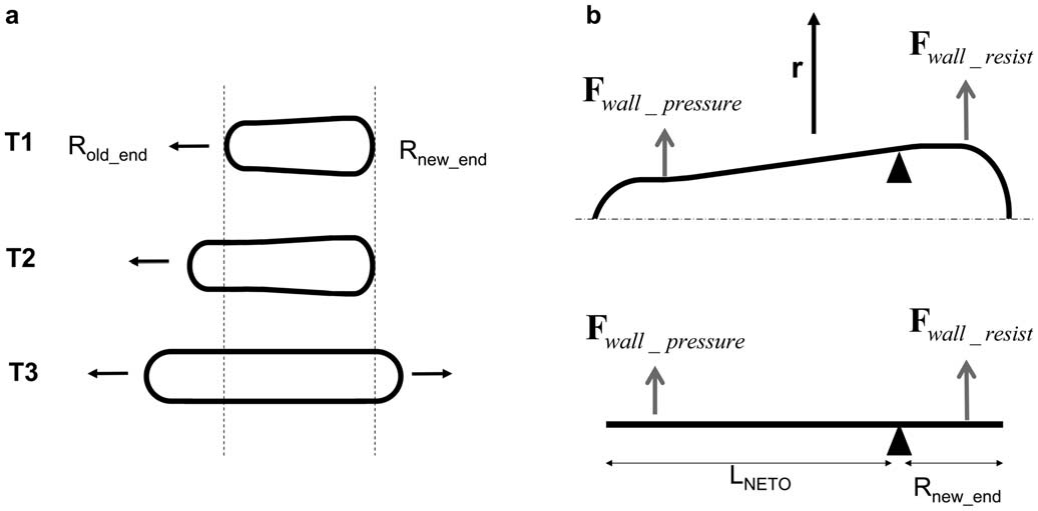
Scaling for NETO length: a/ Right after cytokinesis, only the “old end” elongates (T1 and T2); both radii of curvature Rold_end and Rnew_end are different; above the NETO length, both ends elongate (T3), with similar radii (dotted lines indicate ends locations for a cell attached on a substrate); b/ our equivalent mechanical model (top): the force applied at the old end wall with the lever arm L is opposed by the force of the resisting wall at the new end at a distance Rnew_end; this torque promotes the bending at the new end at NETO; 1-D representation is shown for simplicity (bottom).

After fission, cell growth is monopolar (see Figure 3a). Later in the cycle, above a threshold length, both ends assemble the growth machinery and elongate. This phenomenon was named New End Take-Off (NETO) because the new growing end is elongating only above this length [8]. We propose that NETO is due to a threshold deformation occurring at this new end wall, which reduces the curvature at new end; following my hypothesis, the growth machinery is assembled at this new end, which promotes its elongation.

Several features support this hypothesis: (i) following the Pascal principle, the pressure difference is the same in the cell; as a result, elongation should always occur at both ends; (ii) the old end radius of curvature is smaller than the new end radius of curvature before NETO (see [11], [25], [26]), while having an equal wall thickness (see for example electron microscopy images from Masako Osumi group [21]): the new end appears to be too rigid below a threshold cell length; in contrast, both radii are about the same after NETO.

I propose that the force at the old end triggers mechanically the bending at the new end after NETO, which reduces the radius of curvature at this end. As a consequence, tension is locally reduced, and the growth machinery is recruited locally, as suggested above: this “new end” elongates.

A simple model allows to extract the NETO length above which the radius of the new end decreases. The force associated with the pressure is perpendicular to the wall. As a result, two opposed torques appear along the longitudinal side of the cell at the wall (see Figure 3b). Specifically, two main forces along the radial axis are exerted on the wall at a distance L and Rnew_end respectively of a virtual pivot: the pushing force at the old end
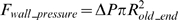
(12)and the elastic force at the new end
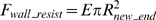
(13)At NETO, I suggest that the torques are equal:
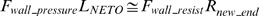
(14)By replacing both forces with their expressions **(12)**and **(13)**, we can write:
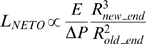
(15)Assuming Rnew_end = 2.2 µm and Rold_end = 2.0 µm, we obtain LNETO∼20 µm.

Note that this model yields the proper order of magnitude for LNETO [8]. A thorough treatment of the model beyond the scope of this paper should allow the derivation of the prefactor for LNETO expression. This scaling law **(15)** could be probed in future experiments with thick mutants (see [27]): the length at NETO should increase with the cell radius.

### The C-shape

**Figure 4:**
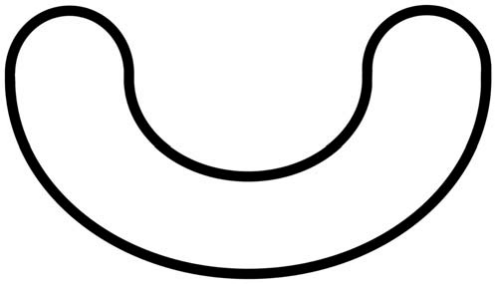
Scaling for a shape mutant: ban mutants exhibit a curved shape, suggesting a buckling phenomenon of the cell wall.

This approach can be used also to explain mutants shapes. For fission yeast, *ban* mutants with a curved shape (Figure 4) were isolated [9]. We propose that the cell wall buckles when a threshold pressure is imposed on the inner wall. We consider fission yeast as a hollow cylinder of inner radius Rin of 1.8 µm and an outer radius Rout of 2.0 µm. The Euler formula gives the maximum axial load that a long, slender, ideal column can carry without buckling [28], [29]. It is set by
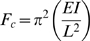
(16)with Fc critical force, E the Young modulus, L the length, and I the geometrical moment of inertia of cross section. This equation can be adapted directly by taking the threshold pressure given by
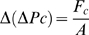
(17)with A the surface of the cell wall under load 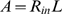. Above this threshold pressure, the cell buckles.

By replacing I by its expression [29], I can estimate the increase in pressure which triggers the cell buckling:
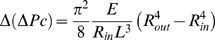
(18)Taking L = 10 µm, E = 100 MPa, Rout = 2.0 µm and Rin = 1.8 µm, we obtain:
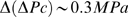



Note that this change in pressure is small compared to my estimate of ΔP = 10 MPa (see Eq. 4). It suggests a fine tuned connection between pressure differences and wall material addition during normal growth. In contrast, a delay in wall addition could cause the observed buckling of the ban mutants.

An experimental set-up similar to the study of microtubule buckling [28], [29] will allow to probe this prediction. By using two pipettes – a rigid one and a flexible one [30]-, a single yeast cell could be held and forced to buckle; by measuring the deflection of the flexible calibrated pipette, our estimate could be checked. In addition, varying the length of the cell undergoing buckling will permit to probe the relation **(18)**: qualitatively, a longer cell will buckle for smaller applied forces.

## Discussion

### The role of molecular mechanisms in this framework

Molecular mechanisms are usually presented for explaining the lengths and shapes of yeast cells [31]. They indeed play a key role in the signalling pathways leading to the read-out observed under the microscope. The same statement applies to the active cytoskeleton: for example, endocytosis at the growing ends via actin mediated transport by patches and filaments [32], the closure of the cytokinetic ring by acto-myosin motors in septum formation [23], or the restrictions of growing ends locations by microtubules [33]. All are involved in the creation of the wall tension. However they are required *intermediates* for assembling the wall and generating tension at the proper locations and phases in the cell cycle, and they do not determine or explain the measured shapes and lengths in a physical sense. The purpose of this work is to suggest coupled approaches where molecular mechanisms in signalling pathways will be characterised simultaneously with the corresponding mesoscopic measurements.

### Conclusion

I have presented scaling arguments for typical read-outs used in fission yeast cellular studies. Similar arguments should hold for other cell types with the appropriate modifications. For example, the wall tension for yeasts is due to its rigid wall whereas the tension for mammalian cells envelope is due to the cortical actin cytoskeleton [34]; in addition, tugor pressure for yeast cells should be replaced by acto-myosin stress in mammalian cells [35], [36], by actin mediated forces in filopodia and lamellipodia [37], or by specific poroelasticity frameworks [38].

The rod shape of fission yeast is important for our arguments, but more than this specific shape, it is its *broken symmetry* which is essential for our reasoning. As a result, our treatment could be extrapolated to other cells. For budding yeast for example, our statement about the difference in surface tension could be used once the bud has emerged [39]. For mammalian cells, similar scaling arguments were tested on the actin cortex remodelling when a broken symmetry was generated [17].

In addition to lengths and shapes of this study, other microscopic read-outs for yeast would follow this logic. For example, it was recently shown that the volumes ratio of nucleus and cytoplasm was conserved in *S. pombe* [40] and in *S. cerevisiae* [41]. This conserved ratio may be derived using laws of chemical physics for dialysis. Altogether this scaling approach for cellular systems should allow to combine microscopic read-outs resulting from signalling networks together with quantitative matter properties.
